# Brain lesions affecting gait recovery in stroke patients

**DOI:** 10.1002/brb3.868

**Published:** 2017-10-25

**Authors:** Kyoung Bo Lee, Joon Sung Kim, Bo Young Hong, Bomi Sul, Seojin Song, Won Jin Sung, Byong Yong Hwang, Seong Hoon Lim

**Affiliations:** ^1^ Department of Rehabilitation Medicine St. Vincent's Hospital College of Medicine The Catholic University of Korea Seoul Korea; ^2^ Department of Physical Therapy Yongin University Yongin Korea

**Keywords:** brain lesion, gait, lesion symptom mapping, recovery, stroke

## Abstract

**Objectives:**

Gait recovery is an important goal in stroke patients. Several studies have sought to uncover relationships between specific brain lesions and the recovery of gait, but the effects of specific brain lesions on gait remain unclear. Thus, we investigated the effects of stroke lesions on gait recovery in stroke patients.

**Materials and Methods:**

In total, 30 subjects with stroke were assessed in a retrograde longitudinal observational study. To assess gait function, the functional ambulation category (FAC) was tested four times: initially (within 2 weeks) and 1, 3, and 6 months after the onset of the stroke. Brain lesions were analyzed via overlap, subtraction, and voxel‐based lesion symptom mapping (VLSM).

**Results:**

Ambulation with FAC improved significantly with time. Subtraction analysis showed that involvement of the corona radiata, internal capsule, globus pallidus, and putamen were associated with poor recovery of gait throughout 6 months after onset. The caudate nucleus did influence poor recovery of gait at 6 months after onset. VLSM revealed that corona radiata, internal capsule, globus pallidus, putamen and cingulum were related with poor recovery of gait at 3 months after onset. Corona radiata, internal capsule, globus pallidus, putamen, primary motor cortex, and caudate nucleus were related with poor recovery of gait at 6 months after onset.

**Conclusion:**

Results identified several important brain lesions for gait recovery in patients with stroke. These results may be useful for planning rehabilitation strategies for gait and understanding the prognosis of gait in stroke patients.

## 1 INTRODUCTION

The restoration of gait is an important goal in stroke patients. Gait regulation and control are complex and are managed evolutionarily by higher centers, with locomotor programming at the level of the cerebral cortex in conjunction with the basal ganglia and the cerebellum (Takakusaki, 2013).

Several studies have investigated the effects of brain lesions on the recovery of gait, and showed that the size of brain lesions affected recovery (Alexander et al., 2009; Kaczmarczyk, Wit, Krawczyk, Zaborski, & Gajewski, 2012). Damage to the posterolateral putamen was associated with temporal gait asymmetry (Alexander et al., 2009). Our previous study showed the caudate nucleus was related to motor recovery in the lower limbs (Lee, Kim, Hong, & Lim, 2017). Another recent study failed to reveal specific lesion locations with regard to balance and gait function (Moon, Pyun, Tae, & Kwon, 2016). Previous researches for Parkinson's disease, s dorsal striatum has been known as a gait pattern generator (Gilat et al., 2017; Peterson, Pickett, Duncan, Perlmutter, & Earhart, 2014; Snijders et al., 2016). However, the role of dorsal striatum for gait recovery is still uncovered in stroke.

Here, we sought to investigate the effects of stroke lesions on gait recovery. Although some studies have demonstrated effects of brain lesions on gait, the effects of specific brain lesions remain unclear. Specifically, we investigated the neurological images and clinical recovery in subjects who had suffered their first supratentorial stroke via lesion symptom mapping. The primary goal of the study was to investigate the effects of stroke lesions on gait recovery in stroke patients.

## 2 MATERIALS AND METHODS

### 2.1 Study design and participants

This study was a retrospective longitudinal, observational clinical trial. Data from 30 right‐handed first‐stroke patients, which were recruited at a single inpatient and outpatient center, from January of 2015 to November of 2015, were analyzed. All subjects had suffered a supratentorial stroke and met the following criteria: (i) first unilateral stroke, (ii) ability to follow verbal instructions, (iii) inability to walk within 2 weeks after stroke, and (iv) a Fugl–Meyer Assessment (FMA) score < 60 for the upper extremities or < 28 for the lower extremities (Sanford, Moreland, Swanson, Stratford, & Gowland, 1993).

Exclusion criteria were (i) presence of knee joint effusion, determined using ultrasound, (ii) a history of knee injury or surgery, or a history of knee injection within 3 months, (iii) a history of inflammatory arthritis or inflammatory myopathy, (iv) diabetes mellitus, or (v) peripheral nervous disease.

From all subjects, we obtained demographic and brain magnetic resonance imaging (MRI) data and evaluated motor and sensory impairments clinically. Brain lesions and sizes were evaluated using a high‐resolution 3 T anatomical MRI with a 5‐mm slice thickness within 14 days after the stroke. This study was an observational one for clinical recovery and outcome, we did not calculate the exact sample size. Sample sizes of previous studies vary 11–41 (Lee et al., 2015; Lee et al., 2017; Shelton & Reding, 2001). We decided the sample size was thirty subjects. Thirty seven patients were enrolled initially, during experimental period. Seven patients were excluded; one patient was excluded for complication as hydrocephalus, one patient was excluded for reattack, five patients were excluded for follow‐up loss (transfer to other rehabilitation centers at a distant location).

All subjects received physical and occupational therapy on the basis of a neurodevelopmental treatment approach (physical therapy) and task‐orientated approach (occupational therapy). The rehabilitation program of all subjects had started within 1 month after onset (Lee et al., 2015). The treatment continued up to 6 months after onset, consisted of 1–2 hrs per day, 5 days a week, including each of physical and occupational therapy. They also received speech therapy (ST), as needed. The interventions were mainly focused on using and strengthening the affected limb, basic mat activity, symmetric weight‐bearing and transfer activities, and gait training, but not performed exclusively for a particular purpose.

The present study protocol was reviewed and approved by the Institutional Review Board of Catholic University, College of Medicine (Registry No. VC16RISE0206). Informed consent was waived by the board.

### 2.2 Clinical evaluations

Clinical tests were conducted with the FMA and functional ambulation category (FAC). Motor and sensory functions were evaluated, using the FMA (Fugl‐Meyer, Jaasko, Leyman, Olsson, & Steglind, 1975). In this study, the score reported did not include the coordination sub‐score (i.e., the highest scores achievable were 60 and 28 for the upper and lower extremities, respectively) (Lee et al., 2015). The ability to ambulate was tested using the FAC (Holden, Gill, & Magliozzi, 1986). The FAC was designed to provide information on the level of physical support needed by the patient to ambulate, both outdoors, and indoors. This assessment included six categories, ranging from 0 (requiring continuous support from two individuals) to 5 (ability to walk indoors and outdoors independently). We considered subjects with FAC scores as ‘three or lower’ to have dependent walking ability. FMA was assessed at the initial assessment (within 2 weeks) for enrollment. FAC assessments were obtained at the initial assessment, and 1, 3, and 6 months postonset.

### 2.3 Lesion tracing and analysis procedures

Lesion locations and size were conducted, using the MRIcron software (http://www.mricro.com/mricron). By using MRIcron software, lesions were mapped on slices of a T2‐weighted MRI scan and were coregistered onto the T1‐weight template MRI scan from the Montreal Neurological Institute (Brett, Leff, Rorden, & Ashburner, 2001; Rorden, Karnath, & Bonilha, 2007). The number of MRI voxels involved in each stroke lesion was calculated. All lesions were traced by a trained image analyst and confirmed by an experienced clinical physiatrist, who was blinded to all clinical data, except for the side of hemiparesis. For a more accurate analysis, the origin of the image (0, 0, 0 mm coordinates) was reoriented such that it was located close to the anterior commissure. The VOI images were transformed to the left hemisphere. To analyze the mutual lesion maps, we used segmentation and normalization (Lee et al., 2015; Sul et al., 2016). The scan and the lesion shape were mapped into stereotaxic space, using the normalization algorithm provided in the SPM8 (http://www.fil.ion.ucl.ac.uk/spm/software/spm8) software. Patients were divided into those able to walk independently and those unable to do so, defined as FAC ≤ 3.

The logic of the subtraction method is straightforward (Lee & Lim, 2017). First, lesions for both groups of patients (dependent vs independent walkers) were placed on the same template image. Next, the lesions for all dependent and independent walker subjects were added together, creating a traditional overlap image, showing regions of mutual involvement. Finally, the lesions for the group of independent walkers were subtracted from the overlap image of the dependent walker group. This process created an image that showed regions that were commonly damaged in patients with dependent walking ability but were spared in the control group. Those of the dependent walker group were coded as positive values and of the independent walker group as negative values.

To avoid potential problems associated with subtraction analysis, we also performed a direct statistical comparison of lesions between able‐walking patient and unable‐walking patient groups, using a voxel based lesion symptom mapping (VLSM) method implemented in the nonparametric mapping (NPM) software included into the MRIcron software (Rorden et al., 2007). Only voxels that were lesioned in at least 10% of all patients were included in the analysis. The nonparametric Liebermeister statistical analysis for binary data was used (Rorden et al., 2007). Colored VLSM maps representing the z statistics were generated and overlaid onto the ch2bet anatomical brain template. The Talairach Daemon software (http://www.talairach.org/) was used to identify the overlaid analysis (Lancaster et al., 2000). Statistical significance was determined as *p *< .05.

### 2.4 Statistical analysis

Data for complete assessments were analyzed, using the SPSS software (ver. 12.0; SPSS Inc., Chicago, IL, USA). Dropout data were not included in the analysis. According to the data distribution, the Friedmann test was used for time‐changes in FAC. If the effect identified, using the Friedman test was significant (*p *< .05), a pair‐wise comparison was performed, using the Wilcoxon's signed‐rank test to identify at which two measurement points a significant difference occurred. Post hoc analyses with the Bonferroni method were used, for which the level of significance was set at *p* < .05. For post hoc analyses of nonparametric statistics, the Bonferroni correction for multiple comparisons was set at *p *< .0167 (i.e., 0.05/3).

## 3 RESULTS

We recruited 30 patients and analyzed (age 55.0±13.7 years; 13 women, 17 men). Of the patients, 15 had left hemiplegia and 15 right hemiplegia. The mean lesion volume was 66328.6±68657.5 voxels (Table 1). Initial motor and sensory functions, assessed by FMA, are shown in Table 1.

**Table 1:** Participant demographics

Demographics (*n *= 30)	
Gender, M/F (%)	56.7/43.3
Age, years^a^	55.0±13.7
Handedness, R/L (%)	100/0
Side of weakness, R/L (%)	50/50
Time from stroke to rehab, days^a^	13.0±6.6
Stroke pathology, hemorrhage/infarction (%)	66.7/33.3
Neglect (%)	23.3
Brain injury location (*n*, %)	
Cortex	2 (6.7)
Subcortex	17 (56.7)
Mixed cortex and subcortex	11 (36.7)
Lesion Volume voxels (*n*)	66328.6±68657.5
Initial motor & sensory^a^	
FM‐UE (0–66)	20.1±18.5
FM‐LE (0–34)	14.0±8.1
FM‐S (0–24)	10.5±8.4

FM‐UE, Fugl–Meyer upper extremity subscore, FM‐LE, Fugl–Meyer lower extremity subscore, FM‐S, Fugl–Meyer sensory subscore.

a Mean ± *SD*.

**Figure 1:**
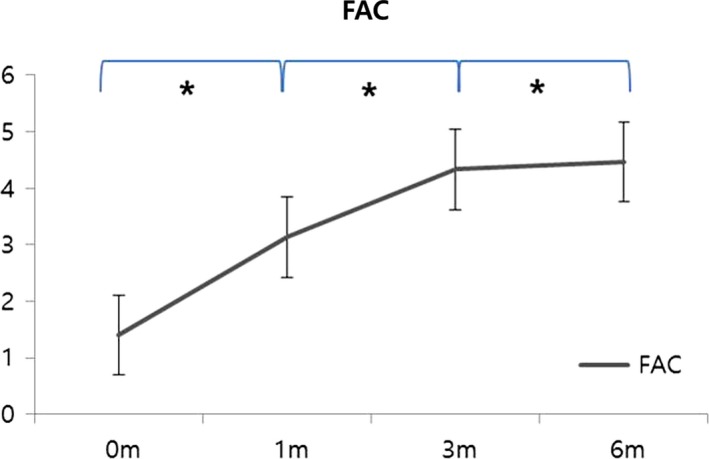
Clinical gait recovery using the functional ambulation category in all subjects (*n *= 30). FAC, Functional ambulation category, * indicates a significant *post hoc* difference (*p* < .0167)

Gait recovery, assessed by FAC with time, is shown in Figure 1. FAC scores improved significantly with time (Figure 1 and table S1).

**Figure 2:**
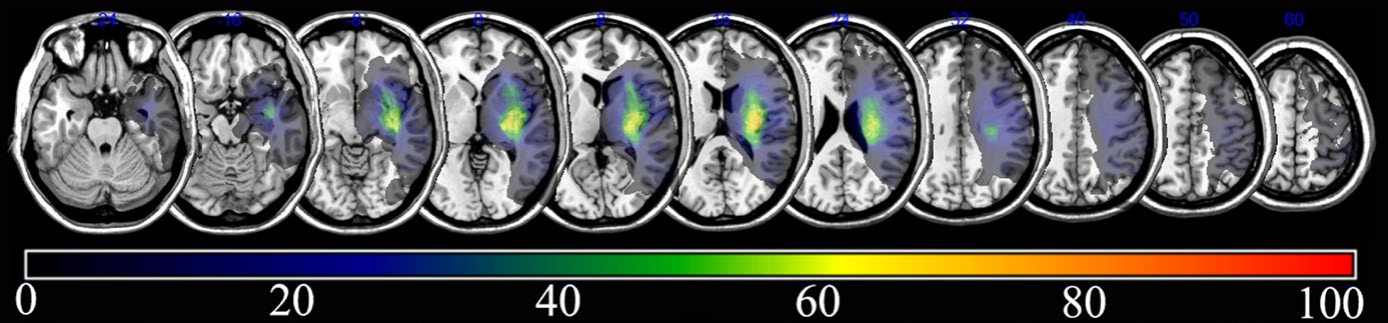
Overlay of lesions in all the subjects with stroke (*n *= 30). The color indicates the frequency of overlap

**Figure 3:**
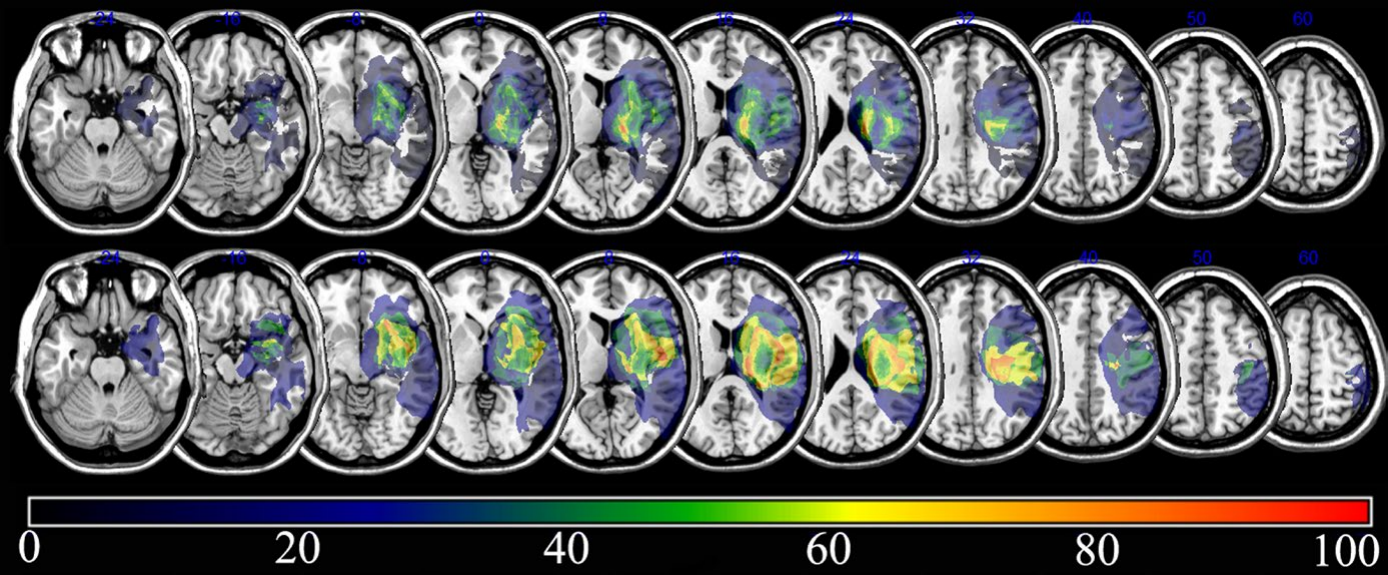
Subtraction analysis, where the overlay of patients without independent walking ability was subtracted from the overlay of those with independent walking ability. The top represents the subtraction analysis where the overlay of patients without independent walking ability was subtracted from the overlay of those with independent walking ability at 3 months post stroke. The bottom represents subtraction analysis where the overlay of patients without independent walking ability was subtracted from the overlay of those with independent walking ability at 6 months post stroke

The overlapping lesions in the brains of all stroke patients included the corona radiata, internal capsule, thalamus, globus pallidus, and putamen (Figure 2; the color represents the frequency of overlap). Subtraction analysis showed that involvement of the corona radiata, internal capsule, globus pallidus, and putamen were related to poor recovery in stroke patients at 3 months after onset. At 6 months after onset, involvement of the corona radiata, internal capsule, and putamen was still important, but the caudate nucleus was also important for gait recovery (Figure 3).

**Figure 4:**
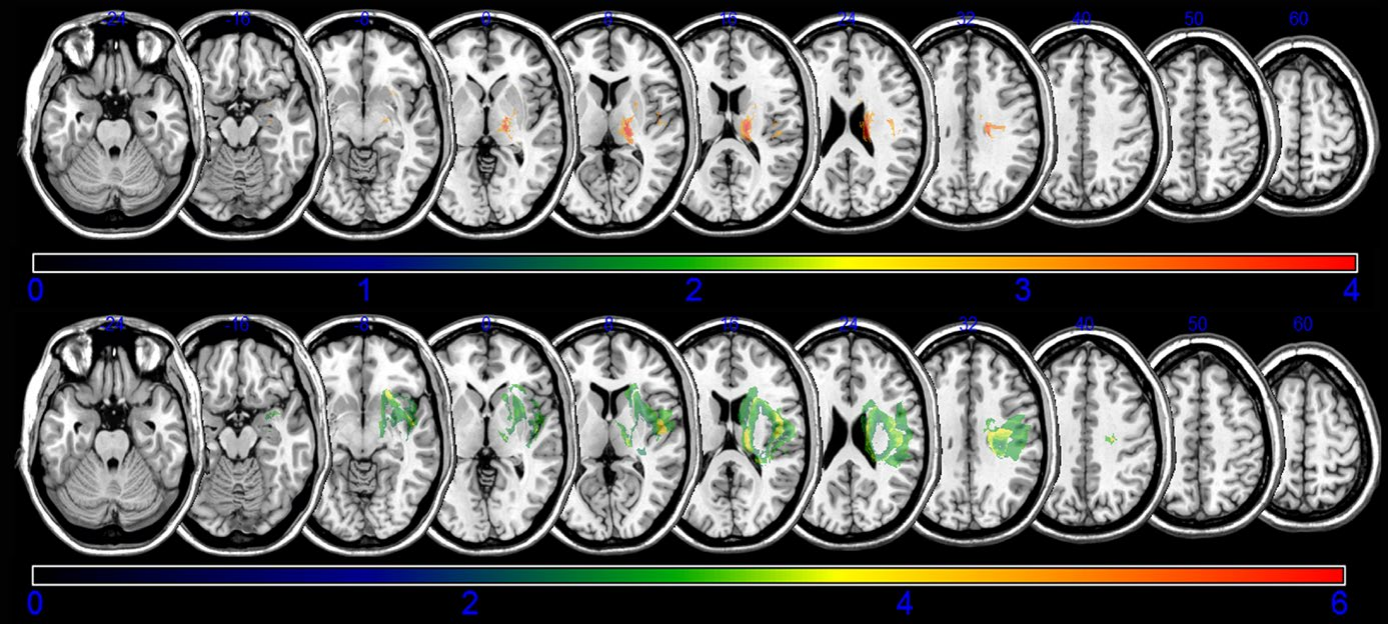
Voxel based lesion symptom mapping for gait. The figure represent direct statistical comparison of lesions between able‐walking patient and unable‐walking patient groups using a voxel based lesion symptom mapping (VLSM) method implemented in the nonparametric mapping software included into the MRIcron software. The top represents VLSM for patients without independent walking ability at 3 months poststroke. We set minimum Z score of 2.96(false discovery rate threshold) and maximum range of the Z score as 4, which be shown as being the maximum brightness. The bottom represents VLSM for patients. Minimum Z score of 1.9740 (false discovery rate threshold) and maximum range of the Z score as 6

Voxel‐based lesion symptom mapping method with nonparametric mapping showed similar results from subtraction analysis (Figure 4). Corona radiate, internal capsule, globus pallidus, putamen, and cingulum were related with poor recovery of gait at 3 months after stroke onset. Corona radiate, internal capsule, globus pallidus, putamen, primary motor cortex, and caudate nucleus were related with poor recovery of gait in stroke patients at 6 months after onset.

## 4 DISCUSSION

Although previous studies demonstrated that several brain lesions were related to the recovery of gait, the effects of specific brain lesions remain unclear. Here, we investigated the effects of stroke lesions on gait recovery in stroke patients, using lesion symptom mapping. Our results suggest that involvement of the corona radiata, internal capsule, globus pallidus, and putamen were related to poor recovery in stroke patients at 3 months after onset. Additionally, injury of the corona radiata, internal capsule, putamen, and caudate nucleus were related to poor recovery of gait at 6 months after the stroke. The VLSM showed very similar results from subtraction analysis of lesion symptom mapping. To our knowledge, this is the first reported longitudinal clinical study to evaluate the effects of brain lesions on gait recovery over a time course with lesion symptom mapping. These observations suggest that injury to the striatum may affect the recovery of gait in stroke patients.

Injury of the posterior corona radiata and posterior limb of the internal capsule may affect motor recovery and functional outcomes (Chen, Tang, Chen, Chung, & Wong, 2000). However, specific brain lesions influencing gait recovery have not been uncovered (Moon et al., 2016). In this study, white matter, such as the corona radiata and internal capsule, apparently affected the recovery of gait throughout the entire observation period, from onset to 6 months after stroke. Several studies have shown associations between the striatum and gait patterns. Injury to the posterolateral putamen was associated with temporal gait asymmetry (Alexander et al., 2009). Caudate nucleus injury was related to motor recovery in the lower limbs (Lee et al., 2017). Neuronal injury in the corona radiata, caudate nucleus, and putamen of patients with chronic stroke changed their walking speed (Jones et al., 2016; Picelli et al., 2014). Our results showed that involvement of the globus pallidus and putamen was related to poor recovery in stroke patients at 3 months after onset. Moreover, involvement of the putamen and caudate nucleus was related with poor recovery of gait at 6 months after the stroke. Thus, the anterior putamen plays a role in the recovery of upper limbs, and the posterolateral putamen has an important role in the recovery of lower limbs (Alexander et al., 2009; Lee et al., 2015). In addition, globus pallidus would be an important structure for gait; it is connected with the cerebellum, the central pattern generator, and plays an inhibitory role while balancing the excitatory effect of the cerebellum in automatic movements (Iosa, Morone, et al., 2016). Our results revealed that globus pallidus had an influence on gait at 3 months after onset upon the VLSM analysis, and at 6 months after onset as well, upon the subtraction analysis. For quality of gait, stroke‐altered latero‐lateral instabilities with the antero‐posterior axis (Iosa, Bini, et al., 2016). Further studies are needed to evaluate the relation between the quality of gait and brain lesion.

Taken together, previous studies and our results show that the striatum has an important role in gait recovery. The pathway among the pedunculopontine nucleus, cerebellum, and the striatum, the supplementary motor area, and the premotor cortex contributes to gait initiation and the modulation of locomotor rhythm and postural muscle tone during locomotion (Gilat et al., 2017; Peterson et al., 2014; Takakusaki, 2013). Thus, injury to the caudate nucleus and putamen is important in gait recovery in stroke patients, especially in the chronic phase(Jenkinson et al., 2009; Takakusaki, 2013). Considering these studies and our results, the determinative lesions for gait recovery seem to differ slightly from the subacute to the chronic phase. At 3 months after onset, the culprit lesions in the brain for gait recovery may be the corticospinal tract itself and the striatum, as the gait pattern generator. At 6 months after onset, the role of the striatum, as the gait pattern generator, was also important in gait recovery. Recent studies of gait training in stroke patients demonstrated that treadmill training with body weight support and traditional gait training were equally effective in improving walking and transfers in patients dependent on assistance in walking after a stroke (Duncan et al., 2011; Hoyer, Jahnsen, Stanghelle, & Strand, 2012). Additionally, repeated gait training, using mechanically assisted walking with body weight support was effective in increasing independent walking in nonambulatory patients (Ada, Dean, Vargas, & Ennis, 2010; Winstein et al., 2016). Traditional rehabilitation therapy (e.g., neurodevelopmental techniques) is an important rehabilitation strategy for gait in patients with stroke, via facilitation or the selective manipulation of sensory information (Wang et al., 2012). In contrast, task‐specific approaches with repeated gait training can facilitate the striatum's role as a gait pattern generator in stroke rehabilitation (Takakusaki, 2013). Our results support the effectiveness of recent rehabilitation programs for task‐specific approaches using, for example, robots or mechanically assisted walking with body weight support (Winstein et al., 2016). Rehabilitation strategies for gait may be more useful and effective based on our results and previous studies.

Because our study has a small number of subjects, we used several focusing methods to overcome bias. We recruited only patients with supratentorial lesions and moderate to severe hemiplegia. This allowed determination of the lesions that are important in gait recovery. In addition, we conducted this study using lesion symptom mapping for gait recovery. Thus, we showed involvement of the striatum, internal capsule, and corona radiata in gait recovery. However, a small number of subjects are still considered an important limitation for clinical implications. Another limitation refers to the starting periods of rehabilitation, which were differing among the subjects, however, this was unavoidable when considering characteristics of retrospective study. Most subjects underwent rehabilitation program within 2 weeks after onset. Rehabilitation of some of the other subjects were delayed, but initiated within 1 month as previously described. Lastly, we did not uncover functional brain network(s) for gait or a specific tract between the striatum and the pedunculopontine nucleus. Further, research for functional network is needed to address these questions.

In conclusion, involvement of the corona radiata, internal capsule, globus pallidus, and putamen were associated with poor recovery in stroke patients at 3 months after onset. Involvement of caudate nucleus was also important for gait recovery at 6 months after onset. These results may be useful for planning rehabilitation strategies for gait and understanding the prognosis of gait in stroke patients.

## CONFLICT OF INTEREST

On behalf of all authors, the corresponding author states that there is no conflict of interest.

## DISCLOSURES

No commercial party having a direct financial interest in the results of the research supporting this article has or will confer a benefit upon the authors or upon any organization with which the authors are associated.
