# Supplementary material for: Brain lesions affecting gait recovery in stroke patients
Source: Brain Behav. 2017 Oct 25;7(11):e00868. doi: 10.1002/brb3.868 (PMC5698874; doi:10.1002/brb3.868)
Supplement: Supplementary file 1 [file BRB3-7-e00868-s001.docx]

**Supplementary Table 1. Change of gait recovery using the functional ambulation category (shown as number(%))**

|  | | 0m | 1m | 3m | 6m |
| --- | --- | --- | --- | --- | --- |
| Dependent walking  (FAC 1-3) | FAC 0 | 17(56.7) | 8(26.7) | 0(0) | 0(0) |
|  | FAC 1 | 5(16.7) | 4(13.3) | 2(6.7) | 0(0) |
|  | FAC 2 | 3(10) | 4(13.3) | 1(3.3) | 1(3.3) |
|  | FAC 3 | 5(16.7) | 4(13.3) | 6(20) | 3(10) |
|  | Sum | 30(100) | 20(66.7) | 9(30) | 4(13.3) |
| Independent walking  (FAC 4-5) | FAC 4 | 0(0) | 5(16.7) | 6(20) | 6(20) |
|  | FAC 5 | 0(0) | 5(16.7) | 15(50) | 20(66.7) |
|  | Sum | 0(0) | 10(33.4) | 21(70) | 26(86.7) |
